# Acetazolamide Mitigates Astrocyte Cellular Edema Following Mild Traumatic Brain Injury

**DOI:** 10.1038/srep33330

**Published:** 2016-09-14

**Authors:** Nasya M. Sturdivant, Sean G. Smith, Syed F. Ali, Jeffrey C. Wolchok, Kartik Balachandran

**Affiliations:** 1 Department of Biomedical Engineering, University of Arkansas, Fayetteville AR 72701, USA; 2 Division of Neurotoxicology, National Center for Toxicological Research, Food and Drug Administration, Jefferson AR 72079, USA

## Abstract

Non-penetrating or mild traumatic brain injury (mTBI) is commonly experienced in accidents, the battlefield and in full-contact sports. Astrocyte cellular edema is one of the major factors that leads to high morbidity post-mTBI. Various studies have reported an upregulation of aquaporin-4 (AQP4), a water channel protein, following brain injury. AZA is an antiepileptic drug that has been shown to inhibit AQP4 expression and in this study we investigate the drug as a therapeutic to mitigate the extent of mTBI induced cellular edema. We hypothesized that mTBI-mediated astrocyte dysfunction, initiated by increased intracellular volume, could be reduced when treated with AZA. We tested our hypothesis in a three-dimensional *in vitro* astrocyte model of mTBI. Samples were subject to no stretch (control) or one high-speed stretch (mTBI) injury. AQP4 expression was significantly increased 24 hours after mTBI. mTBI resulted in a significant increase in the cell swelling within 30 min of mTBI, which was significantly reduced in the presence of AZA. Cell death and expression of S100B was significantly reduced when AZA was added shortly before mTBI stretch. Overall, our data point to occurrence of astrocyte swelling immediately following mTBI, and AZA as a promising treatment to mitigate downstream cellular mortality.

Traumatic brain injury (TBI) is characterized by damage to the brain caused by an external force or blast such as a blow or jolt to the head^1^,^2^. Severity of a TBI increases with increasing force, acceleration, and impact duration^3^, leading to increased contact and acceleration forces experienced by the brain as it is moved by the pressure front. Mild TBI (mTBI), as opposed to moderate or severe TBI, is the most common type of not immediately lethal traumatic brain injury^4^. The term mild implies a reduced severity of the initial trauma, with normal post-trauma brain imaging and minimal cell death compared to moderate or severe TBI, but without precluding downstream pathology^5^,^6^,^7^,^8^,^9^. An estimated 1.7 million TBIs occur annually in the United States with approximately 70% treated in emergency hospitals^1^,^10^,^11^. In particular, the prominent role of improvised explosive devices in the Iraq and Afghanistan wars have led to an increase in the occurrence of TBIs and subsequently an escalation of clinical interest into blast-related TBI^4^,^12^. The primary questions have been how initial cell and tissue-level deformation potentiates overall neuronal and glial dysfunction, and potential therapeutic targets^12^.

Astrocytes outnumber neurons 10:1 and occupy 25% to 50% of the brain volume^13^. Following injury, astrocyte edema or swelling, thus leads to increased intracranial pressures, and is one of the major events that leads to high mortality and morbidity in mild, moderate or severe TBI patients^14^,^15^,^16^. Recent evidence for mechanotransductive astrocyte membrane proteins^17^ as well as the susceptibility of astrocytes to membrane distortions^18^ suggest the potential for astrocytes to respond to pathological mechanical stimuli.

Aquaporin-4 (AQP4) is a highly permeable water channel protein largely expressed in the membranes of astrocytes; particularly those located at the brain-blood and brain-cerebrospinal fluid interfaces^19^,^20^. These water channel proteins play a critical role in the water uptake and regulatory volume decrease of astrocytes during homeostasis^21^,^22^. Since its initial detection in the brain, various studies have shown an upregulation of AQP4 following brain injury, suggesting a possible therapeutic potential for AQP4 antagonists post-injury^23^,^24^,^25^. In this study, we considered the use of the carbonic anhydrase inhibitor acetazolamide (AZA) as a non-specific inhibitor of AQP4 and possible therapeutic for mechanically-induced astrocyte swelling. AZA is an antiepileptic and anti-edema drug that has been proven to inhibit AQP4^26^ as well as reduce brain edema and neuronal death after an intracerebral hemorrhage^27^. AZA can also bypass the blood-brain barrier^28^, increasing its clinical relevance^29^.

We hypothesized that mTBI mechanical forces trigger a swelling response in astrocytes leading to the injury and/or death of these cells. Furthermore, we expect that these harmful responses are positively correlated with the expression of AQP4 channels and can be mitigated when the cells are exposed to the acetazolamide drug prior to mechanical stimuli. To study this hypothesis, we engineered a three-dimensional astrocyte-construct that was subjected to a high-speed mechanical stretch simulating mTBI injury. This construct provided a platform to investigate the effect of mTBI on astrocyte edema, vitality and pathology. In our study, the tissue-engineered astrocyte construct was exposed to AZA 15 minutes before mTBI. Unstretched, non-treated constructs served as controls. The physical mechanism for cell injury was shown to be an acute increase in intracellular volume following mTBI, which was significantly reduced in the presence of AZA. Taken as a whole, our results suggest that inhibition of AQP4 via AZA represents a potential therapeutic strategy for preventing cell swelling after mTBI.

## Results

### *In vitro* mTBI model and validation

**Figure 1:**
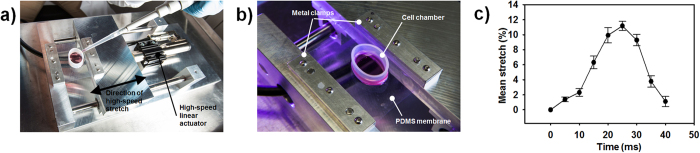
*In vitro* mTBI bioreactor. (**a**) mTBI bioreactor. (**b**) Stretchable cell chamber within mTBI bioreactor. (**c**) Actual mean temporal stretch waveforms within the cell chamber for each mTBI injury cycle.

We sought to develop an *in vitro* mTBI bioreactor that could subject astrocytes in a three-dimensional environment to mTBI injury. Here, we defined mTBI as a magnitude of injury that is sub-threshold for inducing significant cell death, but which did not preclude downstream intracellular injury and pathology^8^,^30^,^31^. A high-speed uniaxial cell stretching device was designed and machined in-house to have control over the stretch and stretch rate. There were two main components to this setup: the cell chamber and the cell stretching device/control system (Fig. 1a). A stretchable cell chamber was made by mounting a 10 mm tall piece of 24.5 mm inner diameter silicon tubing on top of an 8 cm × 7 cm, 250 μm polydimethylsiloxane (PDMS)-membrane (Specialty Manufacturing Inc.). The PDMS-membrane was then braced within metal clamps (Fig. 1b) and mounted to a high-speed linear actuator (Linmot Corp). The cell chamber was prepared by polymerizing 250 μl of Matrigel basement membrane matrix (Corning) within the silicon ring. C8D1A mouse-derived astrocyte cells (ATCC) cultured in standard feed media (Dulbecco’s Modified Eagle Medium (DMEM), 10% fetal bovine serum (FBS), 10 mM HEPES, 1x antibiotic-antimicotic) with 2.5% Matrigel were seeded within the cell chamber as detailed in the Methods Section. Constructs were subject to a single mTBI injury, wherein the astrocyte construct was subject to one cycle of 10% mTBI stretch over the course of 40 ms with constant strain rates (Fig. 1c, Supplementary Video S1). This mode of mechanical loading produced a transient strain field in the tissue, mimicking the *in vivo* tissue deformations during a blow to the head, a fall, or rapid deceleration^8^,^31^. Samples were also subject to treatment with 20 μM of the AQP4 inhibitor acetazolamide^32^ (AZA), 15 minutes prior to mTBI injury. Uninjured samples (0% stretch) without AZA served as controls in this study.

**Figure 2:**
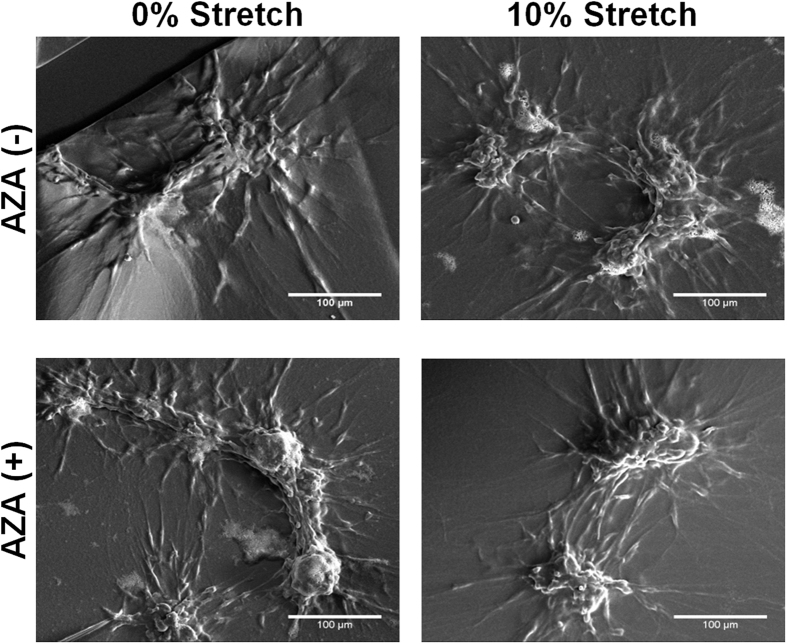
Scanning electron micrographs of astrocyte constructs. Representative scanning electron micrographs depicted no differences in astrocyte construct morphology as a function of the various injury or AZA treatments compared to control untreated samples.

Scanning electron microscopy (SEM) was utilized to study the C8D1A astrocyte construct morphology subject to the two treatments – mTBI injury and AZA (Fig. 2). There was no qualitative difference in cellular morphology. In all the treatment groups, the C8D1A astrocytes were observed to coalesce on top of and within the polymerized layer of Matrigel to form cell clusters approximately 100 μm in diameter, and extending out with projections approximately 100 μm in all directions. The clustering of the cells was observed approximately 24 hours following the stretch (Supplementary Figure S1). We attribute this to the growing confluency of the cells rather than the mTBI stretch. These astrocyte extension processes appeared to extend on top of the Matrigel and also were observed to embed within the construct. Additionally, the C8D1A astrocytes also displayed a characteristic stellate-like morphology, similar to primary cells (Supplementary Figure S1). As the cells formed focal clusters, as opposed to a confluent monolayer sheet, we performed western blots and immunocytochemistry on the C8D1A astrocyte protein lysates to verify that they were still expressing the tight junction protein, Connexin-43 (Supplementary Figure S2).

### AQP4 expression increased after mTBI

AQP4 is a water channel protein known to be involved in astrocyte water homeostasis^33^,^34^. Previous *in vivo* studies indicated that AQP4 expression increased following a TBI^23^,^24^,^25^. We thus first asked if our mTBI model altered AQP4 and whether acetazolamide (AZA), a non-specific AQP4 inhibitor^35^, had any effect on AQP4 expression after mTBI injury.

**Figure 3:**
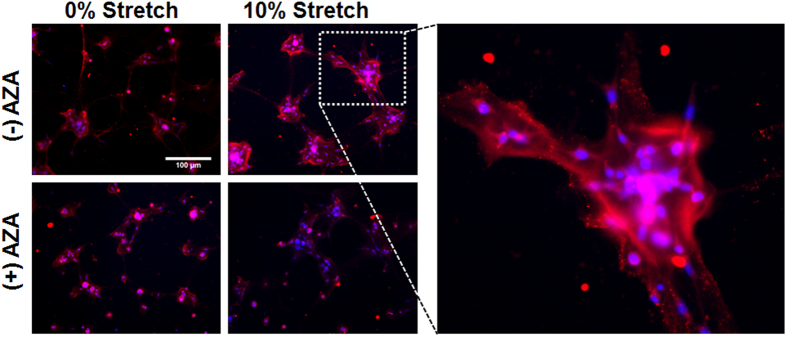
Expression and Localization of AQP4. Representative immunostains of AQP4 expression on C8D1A cells in our *in vitro* model. The cropped and enlarged image on the right shows the localization of the AQP4 expression to the perimeter of the C8D1A cell clusters (endfeet localization).

Immunocytochemistry was performed on the tissue-engineered constructs to detect the presence of AQP4 protein (Fig. 3). AQP4 expression was observed at the perimeter of the C8D1A clusters, indicating that the protein in our mTBI model is indeed localized at the astrocyte endfeet. Delocalization of AQP4 following mTBI is a common occurrence *in vivo.* We did not observe this redistribution in our *in vitro* model (Fig. 3). We acknowledge the lack of redistribution of AQP4 following the mTBI as a weakness of our model. The visibly upregulated expression of AQP4 in stretched constructs indicated that subjecting the cells to an mTBI significantly increased the expression of AQP4 both without and with the presence of AZA. However, as shown in the 10% stretch (+) AZA construct image, adding AZA significantly decreased AQP4 expression that resulted from the mTBI injury. It has been cited that AZA decreases water permeability by physically docking on and blocking the AQP4 channel^36^. Interestingly, in our study we found that the drug decreased AQP4 expression as a whole. The decreased expression of AQP4 in samples that were exposed to AZA suggests that the drug could inhibit cellular water permeability following a mTBI by eliminating AQP4 channels.

**Figure 4:**
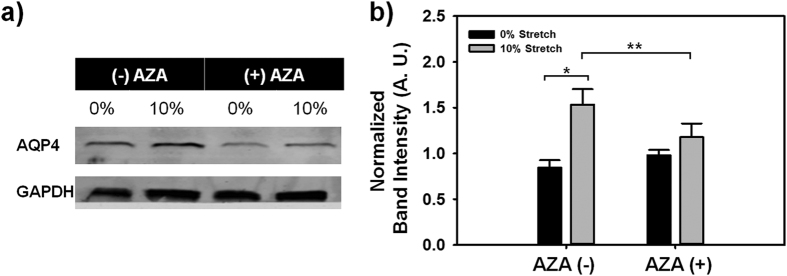
Western blotting analysis of AQP4 expression. (**a**) Representative western blot scans of AQP4 protein expression with GAPDH as control. (**b**) Semi-quantitative analysis of western blotting band intensity (*p < 0.001, **p = 0.051; n = 7).

Western blotting analysis was performed on the tissue-engineered C8D1A astrocyte constructs to further detect the presence of AQP4 (Fig. 4a). Semi-quantitative analysis (Fig. 4b) showed that mTBI significantly increased expression of AQP4 (p < 0.05), in agreement with previous studies^12^,^15^,^16^. AZA had no significant effect on the expression of AQP4 in the uninjured control samples. This is in agreement with recent crystallographic studies showing that AZA inhibited water entry via the AQP4 channel by binding to the AQP4 pore region without potentiating large conformational changes in the AQP4 protein itself^35^,^36^. Overall, the increased expression of AQP4 following mTBI suggests a propensity for the C8D1A astrocytes to increase intracellular water entry via AQP4 following mTBI. Intriguingly, we did not observe any significant difference in AQP4 expression in injured samples treated with AZA, compared to uninjured controls. The effect of mTBI or any mechanical perturbation for that matter, on AQP4-AZA interaction is still poorly understood. This last result suggests a possible alteration in AQP4-AZA interaction after a mechanical insult to the cells.

### Post-mTBI astrocyte swelling is positively correlated with AQP4 expression

**Figure 5:**
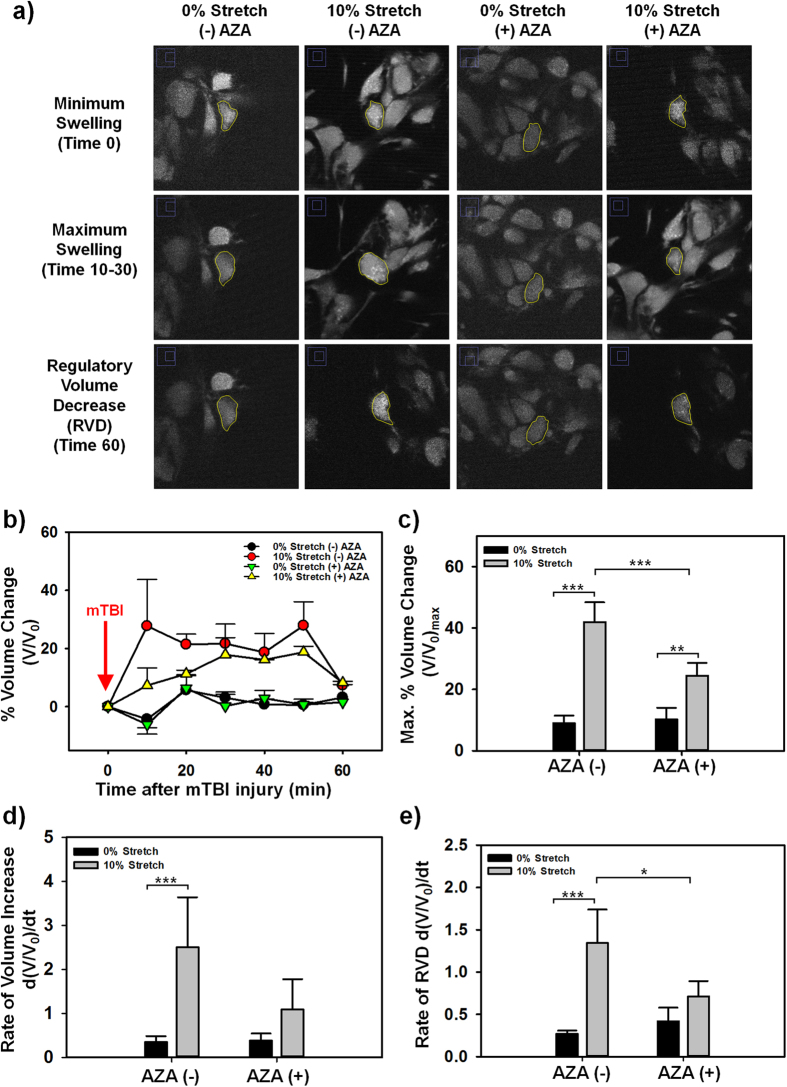
Analysis of astrocyte volume change. (**a**) Representative z-slices showing volume traces of selected cells (outlined with yellow lines). (**b**) Time course of change in astrocyte volume immediately post-mTBI. (**c**) Maximum increase in volume compared to volume at t = 0 (**p = 0.009, ***p < 0.001). (**d**) Rate of cell volume increase following mTBI injury or AZA treatment (***p < 0.001). (**e**) Rate of regulatory volume decrease (RVD) (*p = 0.024, ***p < 0.001).

Previous studies have shown the ability of astrocytes to regulate cell soma volume and cerebral edema due to altered osmolarity via an AQP4-mediated mechanism^37^,^38^. The primary hypothesis of this study was that mTBI injury potentiates astrocyte edema or swelling post-mTBI and that the swelling could be controlled by treating the cells with AZA. While the results from the previous section indicate that AQP4 expression was increased post-mTBI, the following series of experiments were designed to further explore the role of AQP4 in mTBI-induced cell swelling and the potential for AZA to prevent this swelling by asking two questions: I) Was this increase in AQP4 expression directly linked to edema or cell swelling post-mTBI, and II) Can the drug AZA be used to mitigate these responses? Calcein-AM staining and multiphoton z-stack imaging was utilized to quantify the temporal change in volume of C8D1As within the constructs (Fig. 5a). The percent volume change in the cells astrocyte soma volume was significantly (p < 0.05) higher for cells that were injured by mTBI, compared to cells that were not injured, for the entire hour following the stretch (Fig. 5b). Adding AZA to the cells before they were subject to mTBI significantly decreased the percentage increase in cellular volume compared to mTBI injured cells without AZA (Fig. 5b). Uninjured cells (without or with AZA) did not demonstrate any significant change in C8D1A astrocyte soma volume (Fig. 4b). In all groups, the cells demonstrated a return to the original cell volume after approximately an hour, suggesting that the regulatory volume decrease (RVD)^28^ function of the C8D1A astrocytes was not affected by mTBI (Fig. 5b).

Quantification of maximum astrocyte volume increase (Fig. 5c), revealed that mTBI resulted in a 4.6-fold increase in C8D1A astrocyte cell volume after mTBI (p < 0.001). The addition of AZA to constructs that were injured decreased the percentage increased in astrocyte cell volume 1.7-fold (p < 0.001). However, although adding AZA mitigated the effects of the mTBI, there was still a significant difference in cell volume compared to both the uninjured control groups. This result points us to believe that the AZA drug will not cause significant astrocyte volume changes when the cell is in a non-injured state. We also report here a significant difference between the rate at which the soma volume changed as a function of mTBI and AZA treatment. mTBI injury resulted in a significantly higher rate of volume increase (p < 0.001) (Fig. 5d) and a higher rate a of volume decrease (p < 0.001) (Rate of RVD, Fig. 5e) than cells that were not stretched. Adding AZA to the cells had no significant effect on cells that were not injured but it decreased the rate of volume increase and significantly, decreased the rate of RVD in cells that were injured (p < 0.05). If AZA has the ability to slow down brain swelling, this provides additional time for surgical intervention if deemed necessary. Overall, these results suggested that mTBI potentiated acute increases in astrocyte cellular volume that could be mitigated using the drug AZA.

### Astrocyte injury post-mTBI was mitigated by AZA

**Figure 6:**
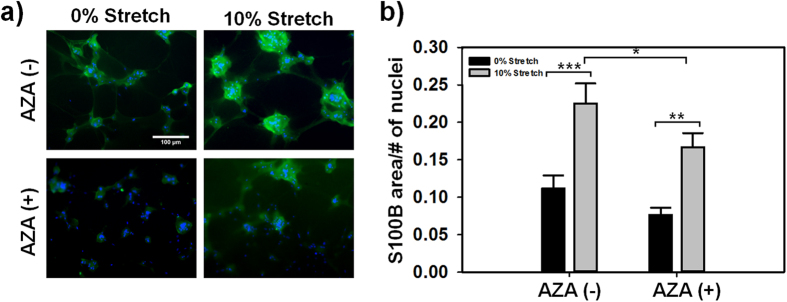
Analysis of S100B expression. (**a**) Representative immunostains of S100B expression. (**b**) Semi-quantitative analysis of S100B immunostains (*p = 0.028, **p = 0.004, ***p < 0.001; n > 18 cells from three separate experiments).

Having demonstrated the effects of AQP4 in regulating C8D1A edema immediately post-mTBI, we next asked if AZA reduced the expression of S100B, a specific marker for injury primarily expressed in astrocytes^39^,^40^, post-mTBI. Immunocytochemistry was performed on the tissue-engineered constructs to detect the presence of S100B protein (Fig. 6a). Semi-quantitative analysis of the images (Fig. 6b) indicated that subjecting the cells to mTBI significantly increased the expression of S100B both without and with the presence of AZA (p < 0.001 and p = 0.004, respectively). However, adding AZA significantly decreased S100B expression that resulted from the mTBI injury (p < 0.05). The expression of S100B was also localized to the astrocyte projections that then extended into the neighboring clusters of cells. The increased expression of S100B in the C8D1A astrocyte constructs that were injured clearly indicates that the mTBI did indeed cause injury to the cells. The decreased expression of S100B in samples that were exposed to AZA suggests that the drug can reduce cell injury severity following mTBI, possibly via its non-specific AQP4-inhibiting mechanism.

### Cell viability post-mTBI increased when pre-treated with AZA

**Figure 7:**
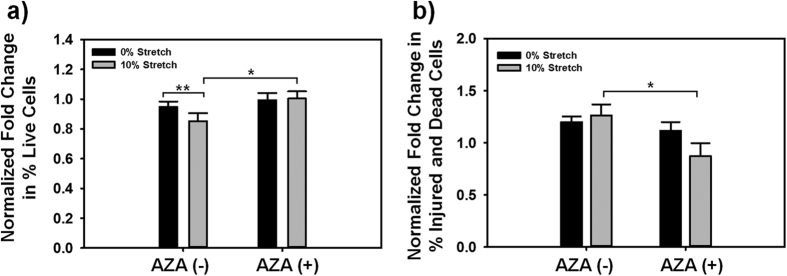
Cell vitality results. (**a**) Normalized fold change of live cells compared to uninjured, untreated controls. (**b**) Normalized fold change of dead and injured cells compared to uninjured, untreated controls. (*p < 0.05, **p = 0.091; n = 7 separate experiments).

While different levels of TBI severity are known to result in varying degrees of cell injury and death, mTBI has traditionally been defined as inducing minimal cellular death^5^,^6^,^7^,^8^,^9^,^41^,^42^,^43^. Therefore, we next asked if mTBI-induced astrocyte edema and expression of injury markers affected cell health and viability 24 hours post-mTBI in our *in vitro* model and whether the AZA mediated these responses. Our results (Fig. 7, Supplementary Fig. S3) demonstrated that there was no significant difference in live cells (Fig. 7a) or injured/dead cells (Fig. 7b) between control (uninjured) and mTBI injured samples, suggesting that our mTBI model did not immediately induce altered cellular viability or death, consistent with previously published definitions of mTBI^5^,^30^,^31^. Compared to the mTBI condition without AZA, cell viability significantly increased (p < 0.05) when AZA was added to the construct before the mTBI injury (Fig. 7a, Supplementary Fig. S3). There were significantly fewer injured and dead cells when AZA was added with mTBI (p < 0.05), compared to cells that were subject to mTBI injury without the AZA treatment (Fig. 7b, Supplementary Fig. S3). These cell vitality results suggest that AZA had a significant effect on cell viability post-mTBI. Taken in context with the cell volume and S100B results, our results suggest that preventing water entry into the cell to cause increased cell volume by inhibiting AQP4 by exposing the cells to the AZA treatment had the beneficial effect of reducing cell death and increasing cell viability post-mTBI.

## Discussion

In this study, we engineered an *in vitro* three-dimensional astrocyte construct and subjected that construct to a uniaxial mTBI to create a benchtop model to investigate acetazolamide (AZA) as a therapeutic option in reducing cell swelling and subsequent cellular injury and death caused by mechanical stimuli. Specifically, we reported two important findings. Firstly, we show that C8D1A astrocytes are mechanosensitive and swell in response to rapid stretch mimicking mTBI injury. Secondly, we demonstrate that the AQP4 antagonist AZA can mute the swelling response, increase cell viability and reduce cell death post-mTBI.

An important distinction between mTBI and TBI or severe TBI is in the severity of downstream cellular and functional responses following the initial injury^8^. In experimental models, the typical definition for mTBI is one in which there is no significant cell death, but detrimental gene expression or functional responses^6^,^7^. Previous work had indicated that the threshold for *in vitro* mTBI was a uniaxial or multiaxial ~10% strain with pulse durations ranging from 25 to 50 ms^5^,^44^,^45^,^46^,^47^. The strain and strain rate parameters utilized in our current study fall within the bounds of these published values. Furthermore, our *in vitro* model benefits from the precise control and the homogenous strain field within the entire engineered astrocyte construct (Fig. 1c), allowing for correlation between observed pathology and input mechanical conditions^48^. The clumpng of cells 24 hours after stretching the network of astrocytes, however, is a limitation of this *in vitro* model; as this is not a characteristic of the glial limitans where the stretching of astrocytes would be most severe. Our mTBI model also resulted in a less than 5% increase in cell death, consistent with previously published reports on mTBI^5^,^30^,^31^.

Edema following TBI or mTBI results in increased patient mortality and long-term pathology and disability^49^. Specifically, cellular swelling, which is the hallmark of cytotoxic edema is known to potentiate signaling cascades that cause profound detriment to cell function^50^. While the exact functional role for AQP4 in mTBI is not well understood^25^, several studies have demonstrated a correlation between AQP4 and increased cellular edema following brain injury^25^,^51^,^52^,^53^,^54^,^55^,^56^,^57^. Our *in vitro* results are in agreement with these *in vivo* findings. Others have also identified oxidative stress^25^ and inflammation^58^ as processes that potentiate increased AQP4 following TBI. In this study, by utilizing AZA as an inhibitor of AQP4 that physically blocks the extracellular water pores in AQP4^36^, we demonstrated reduced C8D1A astrocyte edema and subsequent cellular injury and death after mTBI. Our results therefore suggest that the functional role for AQP4 in post-mTBI pathology is to potentiate increased fluid entry in the cell via its increased expression, thereby causing acute cellular edema. Furthermore, we have clearly demonstrated that blocking the water entry into the cell via AZA inhibition of the AQP4 channel can prevent downstream astrocyte injury and pathology. To our knowledge, our study is the first to report the use of AZA as a therapeutic to reduce astrocyte swelling *in vitro* following a mechanically induced mTBI.

An additional implication of the significant astrocyte swelling observed in our *in vitro* model should be noted. *In vitro*, without any physical constraint on the astrocyte construct, the cells have ample room to swell and the surrounding matrix can rearrange itself to accommodate the increased cellular volume. *In vivo* swelling of the magnitude observed in this study of approximately 40%, due to the physical constraint imposed by the skull, would likely lead to increased intracranial pressures, vascular collapse and potentially ischemic cell death^59^,^60^. Thus, the effects of a similar magnitude of mTBI can be expected to be more detrimental *in vivo*.

As the brain is a highly viscoelastic tissue, the time rates of change in volume or deformation are critical metrics in assessing the pressure response and thus potential pathology post-injury^61^,^62^,^63^. We have reported here a significant increase in rate of C8D1A astrocyte volume increase post-mTBI injury. It is possible that this increased deformation rate could result in increased localized pressure stresses, thus causing increased cellular death and tissue damage. Reducing the rate of deformation or volume change, as we have demonstrated with the AQP4 inhibitor AZA, might mute this pressure spike, therefore allowing the tissue extra time to respond, deform and absorb the pressure stresses over a larger tissue area^61^,^62^,^63^.

Regulatory volume decrease or RVD is a basic cell homeostatic response to altered osmotic or other stresses that cause cellular water entry^64^. In particular, astroglial cells are known to demonstrate a very rapid RVD response^65^. We demonstrate here that the RVD response of C8D1As was accelerated during mTBI, possibly as a compensatory mechanism to recover from the increased intracellular edema post-mTBI. However, although C8D1A astrocytes subjected to mTBI were able to recover their initial volume within an hour following injury, the downstream injury cascade was not mitigated. The presence of AZA significantly reduced the rate of RVD as well as downstream injury, suggesting that the initial magnitude of cell volume increase was the critical factor in potentiating astrocyte injury post-mTBI.

In summary, we demonstrated that mTBI injury resulted in altered AQP4 expression that potentiated acute increases in intracellular volume, increased cellular S100B expression and injury. Our study helps to elucidate the key role the AQP4 channel plays in cell edema and death following mTBI and provides a possible therapeutic option, via AZA pre-treatment, for minimizing cell swelling caused by mTBI. Our work also provides an important foundation for the future studies on the treatment of mTBI-induced injury in astrocytes with AQP4-inhibitors.

## Methods

### *In vitro* mTBI model and validation

A cell stretching device was designed and machined to have control over the stretch and stretch rate. There were two main components to this setup: the cell chamber and the cell stretching device/control system (Fig. 1a). A stretchable cell chamber was made by mounting a 10 mm tall piece of silicon tubing on top of an 8 cm × 7 cm, 0.010” polydimethylsiloxane (PDMS)-membrane. The PDMS-membrane was then braced within metal clamps (Fig. 1b). Chamber parts were sterilized using an ethylene oxide sterilizer (Anprolene). For validation of mTBI stretch injury, we first spread 250 μL of Corning Matrigel Basement Membrane Matrix evenly within the cell chamber, polymerized the Matrigel for 20 minutes at 37 °C, and marked the top of the gel with a 4 × 6 point grid (Supplementary Fig. S1). The chamber was mounted on to the stretching device and we recorded the displacement of the marker grid during mTBI programmed injury using a Basler a640 area-scan camera at 200 frames per second (Supplemental Video S2). The programmed input strain for mTBI injury was a peak strain of 10% in 40 ms. Using a custom-written MATLAB script, we tracked the motion of the marker grid and calculated the mean strain within each element of the grid (Fig. 1c).

### Cell culture

Preliminary culture of the mouse-derived C8D1A [Astrocyte type I clone] cell line(ATCC^®^ CRL 2541™) was done in standard feed media (Dulbecco’s Modified Eagle Medium (DMEM), 10% fetal bovine serum (FBS), 10 mM HEPES, 1x antibiotic-antimicotic) at 37 °C and 5% CO_2_. Prior to seeding of the cells, PDMS chambers were further sterilized and activated with UV-Ozone for 20 minutes. To prepare samples for mTBI injury, 250 μL of Corning Matrigel Basement Membrane Matrix was evenly spread within the stretchable chamber and set in the 37 °C incubator for 20 minutes to polymerize. 500,000 C8D1A astrocytes were then dissociated from preliminary culture, resuspended in feed media/2.5% Matrigel and seeded on top of the polymerized layer of Matrigel to create a three-dimensional culture. The tissue-engineered astrocyte construct was incubated at 37 °C for 24 hours to allow proper time for a semi-confluent network of astrocytes to form on the Matrigel before being subjected to either mTBI injury or control treatment (no injury).

We also separately isolated and cultured primary rat astrocytes from neonatal day 1 rat pups obtained from timed pregnant Sprague Dawley rats, utilizing published methods^66^. All animal experiments were approved by the University of Arkansas Institutional Animal Care and Use Committee, and performed in accordance with the relevant guidelines and regulations.

### Cell Swelling Treatment

20 μM of the non-specific AQP4 inhibitor acetazolamide^32^ (AZA) (Sigma-Aldrich, St. Louis, MO) was used to regulate water passage into the cell and control cell swelling post-mTBI. AZA was added to the media 15 minutes before stretch. We then maintained the cell constructs in contact with AZA until the end-point of that experiment, including any PBS washes as well as cell recovery solutions.

### Sample preparation for scanning electron microscopy and imaging

Scanning electron microscopy (SEM) images were taken for assessment of cell attachment and morphology. Samples were washed twice with Dulbecco’s phosphate buffered saline (PBS) and once with sodium cacodylate buffer. The samples were then fixed in 2.5% PFA/2.5% glutaraldehye in sodium cacodylate buffer for 2 hours at 4 °C. Following fixation, the cells were washed again in sodium cacodylate buffer three times and completely dehydrated with a series of ethanol washes (50%, 75%, 95%, 100%). Once completely dry, the samples were sputter coated with gold and viewed using a FEI™ Nova Nanolab 200 scanning electron microscope.

### Isolation of C8D1A astroctyes from Matrigel

After appropriate treatment, the astrocytes were isolated from the Matrigel before performing proteomic analysis. Briefly, 24 hours after stretch the astrocyte constructs were washed 2X with PBS and scraped out of the chamber into a centrifuge tube with Corning Cell Recovery Solution. The cells were incubated in the Recovery Solution for 1 hour on ice, to allow the cells enough time to separate from the Matrigel. After this period, the samples were centrifuged at 300 g for 5 minutes and then the Matrigel and Cell Recovery Solution supernatant were carefully removed from the cells. The remaining C8D1A cells were analyzed as outlined in sections below.

### Immunostaining

C8D1A/Matrigel samples were immunostained with antibodies against DAPI, S100B, AQP4, and Connexin 43. Briefly, twenty-four hours after injury, the samples were rinsed three times with 1X PBS and fixed in 4% paraformaldehyde for 20 minutes, followed by three more rinses with PBS. 20% goat serum was then pipetted onto the cells, for blocking, and incubated at 37 °C for 1 hour. Next, anti-AQP4 antibody (Abcam, ab46182), anti-S100B antibody (Abcam, ab52642), or anti-Connexin 43 (Abcam, ab11370) used at a dilution of 1:50 (AQP4 and S100B) or 1:1000 (Connexin 43) with 2% goat serum, was pipetted onto the gels and allowed to incubate overnight in 4 °C. Primary antibody solution was aspirated and the gels were rinsed 3 times with PBS for 15 min each. Secondary antibody solution comprised of 4′,6-diamidino-2-phenylindole (DAPI) to stain cell nuclei and donkey anti-rabbit 594 or donkey anti-rabbit 488; all used at a dilution of 1:200. Secondary stain was left on and protected from light for 1–2 hours. Following removal of the secondary antibody solution and another rinse cycle, the cells were imaged using a Nikon Eclipse epifluorescent microscope at a 20X magnification. S100B fluorescent images were analyzed semi-quantitatively with the use of ImageJ. Results were presented as a ratio of antibody coverage area normalized by the number of nuclei per image field. We obtained at least 8 image fields from three separate samples for this analysis.

### Western Blotting

After isolation from Matrigel, the astrocytes were lysed with lysis buffer (4 M urea, 5 mM EDTA, 0.5% SDS, 0.5% NP-40, 100 mM Tris pH 7.4) containing protease and phosphatase inhibitor (Sigma-Aldrich, St. Louis, MO). The homogenates were centrifuged at max speed for 5 minutes at 4 °C. Cell debris was withdrawn leaving just the supernatants followed by determination of protein concentration with a BCA protein assay. 40 μg/lane of protein was loaded onto a Criterion TGX Precast gel and subsequently transferred onto PVDF (polyvinyl difluoride) membrane. Following transfer, the membranes were blocked with Odyssey Blocking Buffer (LI-COR, Bioscence, Germany). The membranes were then probed with Rabbit anti-AQP4 antibody (Abcam, ab46182), anti-Connexin 43 (Abcam, ab11370) and Mouse anti-GAPDH (Abcam, ab8245) in Odyssey Blocking Buffer. GAPDH was used as the gel loading control. Both the AQP4 and GAPDH antibodies were used at a dilution of 1:500. Secondary antibodies (donkey anti-rabbit 800 and donkey anti-mouse 700) were used at a dilution of 1:20,000. After Western blotting, the protein bands were detected with a Li-cor Odyssey infrared scanner. Band integrated optical density was quantified using ImageJ software. Sample size was at least 5 for each treatment group.

### Astrocyte volume analysis with calcein staining

Just prior to appropriate treatment, the cell constructs were washed with PBS and stained with 10 μM calcein AM for 45 minutes. The constructs were then incubated with Tyrode’s buffer and subject to appropriate mTBI or control. Immediately after, the cells were analyzed under a multi-photon microscope. A 40× (0.80 NA) water immersion lens with a 2x additional zoom was used to image the C8D1A astrocytes. Excitation wavelength was 980 nm and emission was collected using a 525/50 nm emission filter. Z-stack images with 0.3 μm z-slice thickness were acquired every 10 minutes for 1 hour for volumetric analysis. Cell volumes were assessed by importing a z-stack into a custom MATLAB script. The script allowed for the manual tracing of the cell perimeter and subsequent calculation of the area of the cell in that particular z-slice. The areas of the cell at each slice in the stack were used to compute the volume of that particular cell. At least 6 cells were analyzed for each treatment group from at least 3 separate experiments.

### Cell vitality assay

Astrocyte vitality following mTBI injury was assessed using a commercially available cytotoxicity assay (LIVE/DEAD Cell Vitality Assay Kit, C12-Resazurin/SYTOX Green; Molecular Probes, Eugene OR) and flow cytometry. First, the tissue-engineered constructs were rinsed 3 times with cold PBS 24 hours following appropriate treatment. The cells were then isolated from the Matrigel with the use of Corning Cell Recovery Solution as outlined earlier. After isolation from the Matrigel, astrocytes were resuspended in PBS and incubated with 4 μL of 50 μM C12-resazurin working solution and 4 μL of 1 μM SYTOX Green working solution for 30 minutes at 37 °C. C12-resazurin is reduced to a red-fluorescent C12-resorufin in metabolically active cells. Cells with a comprised plasma membrane, usually seen in apoptotic and necrotic cells, uptake the green-fluorescent nucleic stain, SYTOX Green dye. Cells with a damaged membrane but with a low level of metabolic activity uptake, emitted a reduced level of SYTOX Green and red fluorescence and were classified as injured cells. We can thus partition cells as live, injured or dead based on their uptake of the aforementioned dyes (Supplementary Fig. S1). Cell fluorescence was then acquired using a BD FACSCanto II flow cytometer and analyzed using FlowJo (Ashland, Oregon). SYTOX green and C12-resorufin fluorescence were collected on the fluorescein isothiocyanate and R-phycoerythrin channels respectively. The percentage of live, injured, and dead cells present following the various treatments were normalized with the control (0% stretch, No AZA) and presented.

### Statistical analysis

Data was first tested for normality using the Shapiro-Wilk test. Statistical analysis was carried out by two-way analysis of variance (ANOVA) for normally distributed data. Non-parametric analysis was used for data that was not normally distributed. SigmaPlot software was used for the statistical analysis. Values were expressed as mean ± standard error of the mean (SEM). A p-value less than 0.05 was considered significant and denoted with a single asterisk (*).

## Additional Information

**How to cite this article**: Sturdivant, N. M. *et al*. Acetazolamide Mitigates Astrocyte Cellular Edema Following Mild Traumatic Brain Injury. *Sci. Rep.* **6**, 33330; doi: 10.1038/srep33330 (2016).

## Supplementary Material

Supplementary Video 1

Supplementary Video 2

Supplementary Information
