# Supplementary figures and images for: Acetazolamide Mitigates Astrocyte Cellular Edema Following Mild Traumatic Brain Injury
Source: Sci Rep. 2016 Sep 14;6:33330. doi: 10.1038/srep33330 (PMC5022024; doi:10.1038/srep33330)

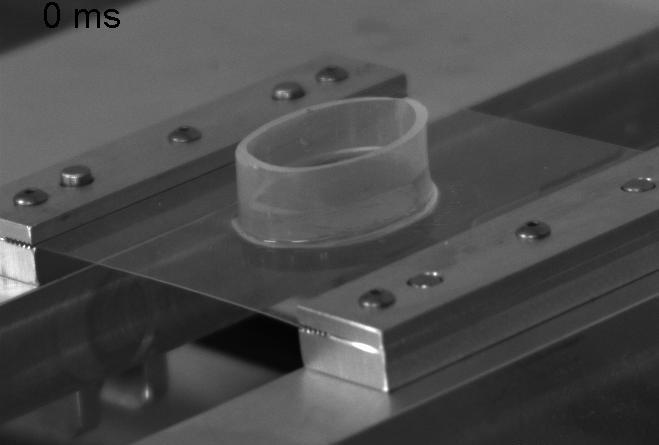

Supplement: Supplementary Video 1 [file srep33330-s1.gif]

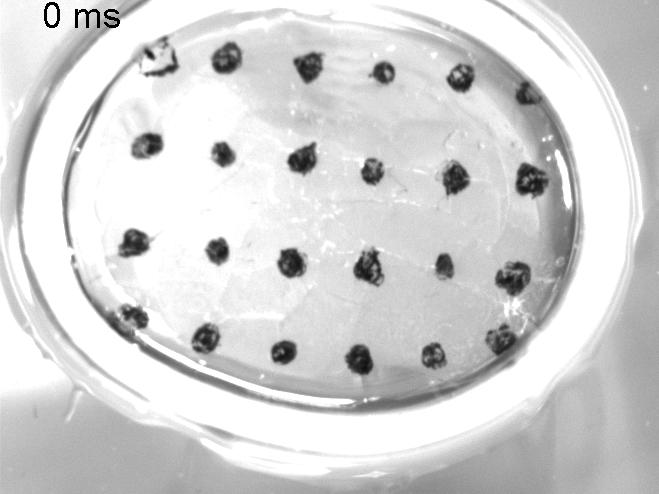

Supplement: Supplementary Video 2 [file srep33330-s2.gif]
