# Supplementary material for: Acetazolamide Mitigates Astrocyte Cellular Edema Following Mild Traumatic Brain Injury
Source: Sci Rep. 2016 Sep 14;6:33330. doi: 10.1038/srep33330 (PMC5022024; doi:10.1038/srep33330)
Supplement: Supplementary Information [file srep33330-s3.doc]

**Acetazolamide Mitigates Astrocyte Cellular Edema Following Mild Traumatic Brain Injury**

Nasya M. Sturdivant^a^, Sean G. Smith^a^, Syed F. Ali^b^, Jeffrey C. Wolchok^a^ and

Kartik Balachandran^a^

^a^Department of Biomedical Engineering, University of Arkansas, Fayetteville AR 72701

^b^Division of Neurotoxicology, National Center for Toxicological Research, Food and Drug Administration, Jefferson AR 72079, USA

**Corresponding author:**

Kartik Balachandran, Ph.D.

Assistant Professor
Department of Biomedical Engineering
122 John A. White Jr. Engineering Hall

Fayetteville, AR 72701

Email: [kbalacha@uark.edu](mailto:kbalacha@uark.edu)

**Supplementary Information**

**
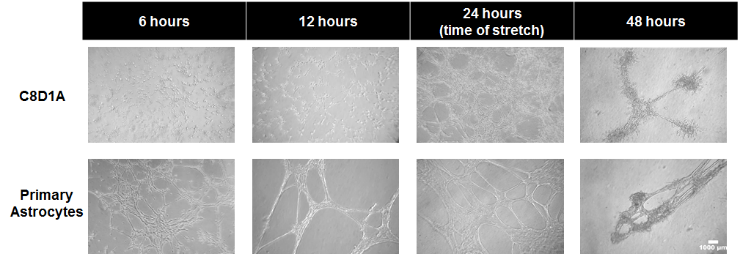
**

**Supplementary Figure S1.** **Representative brightfield images of C8D1A astrocyte cell line and primary astrocytes.** The primary astrocytes were isolated from the brain of neonatal rats. Images were taken 6, 12, 24, and 48 hours after being seeded on the Matrigel. (Scale bar 1000 µm)

**
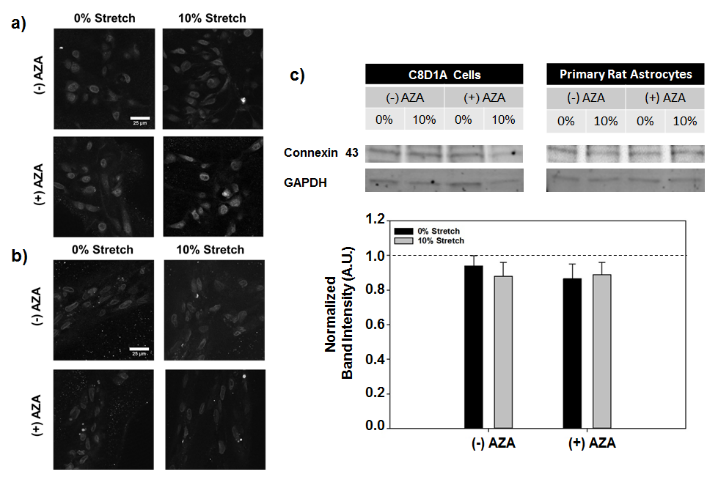
**

**Supplementary Figure S2. Comparable expression of Connexin 43 in C8D1A cells and primary astrocytes.** (a) Representative immunostains of Connexin 43 expression in C8D1A cells, (b) Representative immunostains of Connexin 43 expression in primary astrocytes Scale bar 25 µm), and (c) Representative western blot scans and the semi-quantitative analysis of Connexin 43 protein expression western blotting band intensity. Reported here are the C8D1A band intensities normalized to the primary rat astrocyte band intensities (n=6).

**
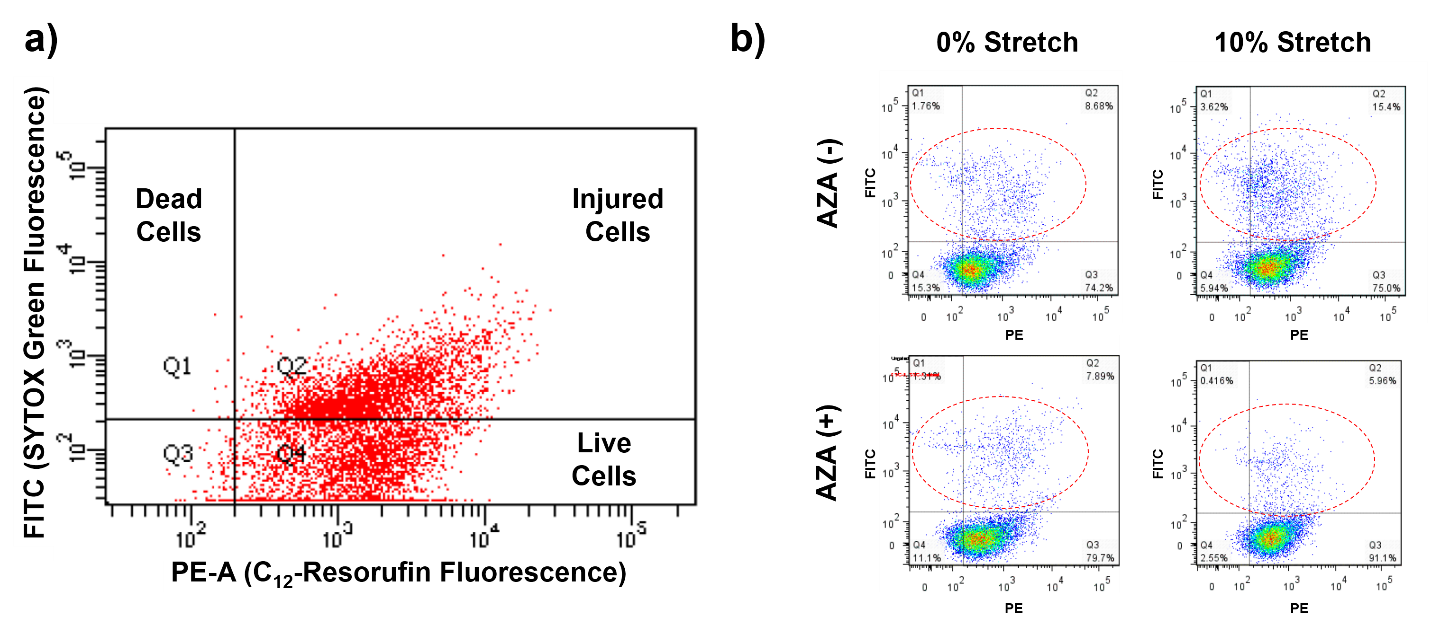
**

**Supplementary Figure S3. Cell Vitality Assay Flow Cytometry Data.** (a) Representative scatter plot of one flow cytometry acquisition from a 10% stretch mTBI injured sample, demonstrating the partitioning of live, injured and dead cell populations. (b) Representative data acquired for uninjured (0% stretch) and mTBI injured (10% stretch) samples without and with AZA. Red dotted circle denotes dead and injured fraction. Note increased number of dead and injured cells in mTBI injured sample without AZA.

**
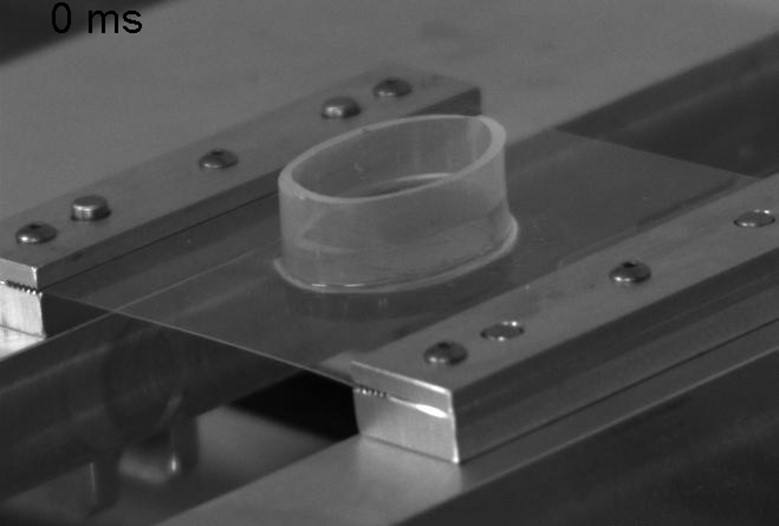
Supplementary Video S1.** Snapshot from representative video demonstrating one cycle of high-speed stretch used to mimic mTBI. Video included as supplementary material.


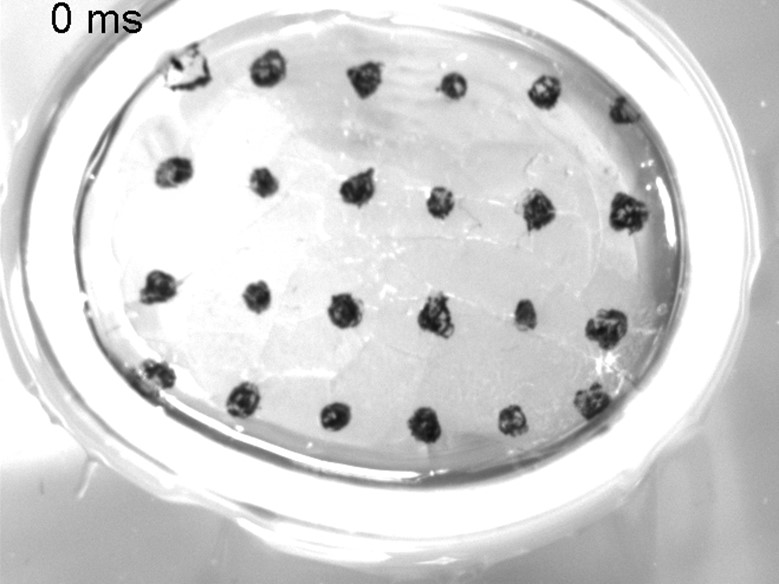


**Supplementary Video S2.** Snapshot from representative video showing marker grid used to validate strain on Matrigel construct. Video included as supplementary material.
